# Artesunate-amodiaquine versus artemether-lumefantrine for the treatment of acute uncomplicated malaria in Congolese children under 10 years old living in a suburban area: a randomized study

**DOI:** 10.1186/s12936-015-0918-6

**Published:** 2015-10-29

**Authors:** Mathieu Ndounga, Pembe Issamou Mayengue, Prisca Nadine Casimiro, Félix Koukouikila-Koussounda, Michel Bitemo, Brunelle Diassivy Matondo, Lee Aymar Ndounga Diakou, Leonardo K. Basco, Francine Ntoumi

**Affiliations:** Unité de Recherche sur le Paludisme, Centre d’Etudes sur les Ressources Végétales (CERVE), BP 1249, Brazzaville, Republic of Congo; Fondation Congolaise pour la Recherche Médicale (FCRM), BP 2672, Brazzaville, Republic of Congo; Institut de Recherche pour le Développement (IRD), Unité Mixte de Recherche 198, Unité de Recherche sur les Maladies Infectieuses et Tropicales Emergentes (URMITE), Faculté de Médecine La Timone, Aix-Marseille Université, Marseille, France; Institute for Tropical Medicine, University of Tübingen, Tübingen, Germany; Faculté des Sciences et Techniques, Université Marien Ngouabi, BP 69, Brazzaville, Republic of Congo; Laboratoire National de Santé Publique, BP 120, Brazzaville, Republic of Congo

**Keywords:** Drug resistance, Artemisinin-based combination therapy, Sickle cell trait, Drug efficacy, Congo-Brazzaville

## Abstract

**Background:**

The Republic of Congo adopted a new anti-malarial treatment policy in 2006, with artesunate-amodiaquine (ASAQ) and artemether-lumefantrine (AL) as the first- and second-line anti-malarial drugs, respectively. Only three clinical studies had been conducted before the policy change. A randomized study on these two artemisinin-based combinations was conducted, and the effect that sickle cell trait may have on treatment outcomes was evaluated in children under 10 years old followed during 12 months in Brazzaville in 2010–2011.

**Methods:**

A cohort of 330 children under 10 years of age living in a suburban area in the south of Brazzaville were passively followed for registration of malaria episodes. Uncomplicated *Plasmodium falciparum* episodes were randomly treated with co-formulated ASAQ (Coarsucam^®^) or AL (Coartem^®^). Patients were followed according to the 2009 World Health Organization protocol for the evaluation of anti-malarial drug efficacy. *Plasmodium falciparum* recrudescent isolates were compared to pre-treatment isolates by polymerase chain reaction (PCR) to distinguish between re-infection and recrudescence. PCR-uncorrected and PCR-corrected responses to treatment were determined using per protocol analysis. Haemoglobin type (AA, AS, SS) was determined by PCR.

**Results:**

Of 282 clinical malaria episodes registered during 1-year follow-up period, 262 children with uncomplicated malaria were treated with ASAQ (129 patients) or AL (133 patients). The PCR-corrected efficacy, expressed as the percentage of adequate clinical and parasitological response, was 97.0 % for ASAQ and 96.4 % for AL. Among ASAQ-treated patients, 112 (86.8 %) carried AA genotype and 17 (13.2 %) were AS carriers. The PCR-corrected efficacy was 96.4 % for AA-carriers and 100 % for AS-carriers treated with ASAQ [relative risk (RR) = 0.96; 95 % confidence interval, 0.93–1.00, p = 0.5]. Among 133 AL-treated children, 109 (82 %) carried AA, and 24 (18 %) AS genotypes. The PCR-corrected efficacy was 96.7 % among AA-carriers and 95.2 % among AS-carriers [RR = 1.01 (0.92–1.12), p = 0.6]. Nausea, jaundice, headache, dizziness, vomiting, pruritus, abdominal pain, and diarrhoea were registered as adverse events in both groups. ASAQ was associated with significantly more frequent adverse events (P < 0.05).

**Conclusion:**

This first randomized study in Brazzaville confirmed the excellent efficacy of these co-formulated anti-malarial drugs in children. Sickle cell genotype did not influence the treatment efficacy of artemisinin-based combination therapy.

## Background

In the 1990s, with the emergence and spread of chloroquine resistance, malaria accounted for 37 % of hospitalizations and represented the first cause of admissions to paediatric service of the main hospital in Brazzaville, Republic of Congo (RoC) [1]. A significant increase in the cases of cerebral malaria leading to death was also observed [2]. In health facilities situated in urban areas, 15–30 % of febrile patients had been found to be carriers of malaria parasites, while in suburban areas, 40–50 % of patients were infected [3].

4-aminoquinolines and quinoline-like derivatives (chloroquine, amodiaquine) and sulfadoxine-pyrimethamine (SP) have lost their efficacy for the treatment of uncomplicated falciparum malaria in RoC [4, 5]. Indeed, almost all of *Plasmodium falciparum* isolates analysed in earlier studies carried the key *pfcrt* K76T mutation associated with chloroquine resistance, while quadruple mutations [defined as dihydrofolate reductase (*dhfr*) mutant alleles Asn51Ile + Cys59Arg + Ser108Asn and dihydropteroate syntase (*dhps*) mutant allele Ala437Gly] were present in more than 50 % of the isolates obtained from patients with SP treatment failure [6, 7]. Under these conditions, the management of malaria in health centres and at home had become difficult with anti-malarial drugs available in the early 2000s [8].

Artemisinin derivatives (artesunate, artemether, dihydroartemisinin) in combination with amodiaquine, sulfadoxine-pyrimethamine, lumefantrine, mefloquine, piperaquine, or pyronaridine have largely replaced chloroquine and sulfadoxine-pyrimethamine as effective alternative drugs. In the early 2000s, the World Health Organization (WHO) strongly recommended a change of malaria treatment policies based on artemisinin-based combination therapy (ACT) [9, 10]. By 2006, 36 African countries had changed their policies, but only 18 implemented their new policies [11], while in 2011, ACT was available in all African countries [12]. The progressive introduction of ACT and vector control measures have significantly reduced the prevalence of clinical cases and mortality due to malaria [13, 14].

In 2006, RoC adopted a new treatment policy for uncomplicated malaria with artesunate-amodiaquine (ASAQ) and artemether-lumefantrine (AL) as the first- and second-line drugs, respectively [15]. Three clinical studies were conducted before the decision: one randomized study in a rural area near Brazzaville [16] and two non-randomized studies in Brazzaville [17, 18]. In these three published studies, non-co-formulated drugs were administered to symptomatic patients. Due to the limited data on the clinical efficacy of ACT, further clinical studies are required to cover the rest of the country to monitor the efficacy of ASAQ and AL using co-formulated drugs.

It has been observed that anti-malarial treatment failures may be caused by many factors other than the intrinsic susceptibility of *P. falciparum* to the anti-malarial drug being tested [19]. Therefore, an understanding of the different factors that influence the clinical and parasitological response is important for the correct interpretation of estimates of drug efficacy. There are several studies suggesting that host genetic factors play a role in determining anti-malarial treatment outcome, including evidence for an increased effect of sulfadoxine-pyrimethamine against *P. falciparum* in the presence of the sickle cell trait (HbAS) or a reduced efficacy of chloroquine and artemisinin derivatives in patients with α-thalassaemia [20–23].

This is the first randomized trial conducted in Brazzaville between 2010 and 2011 in which the efficacy of co-formulated ASAQ and AL and the implication of the sickle cell trait on treatment outcomes were evaluated.

## Methods

### Study site and population

**Fig. 1:**
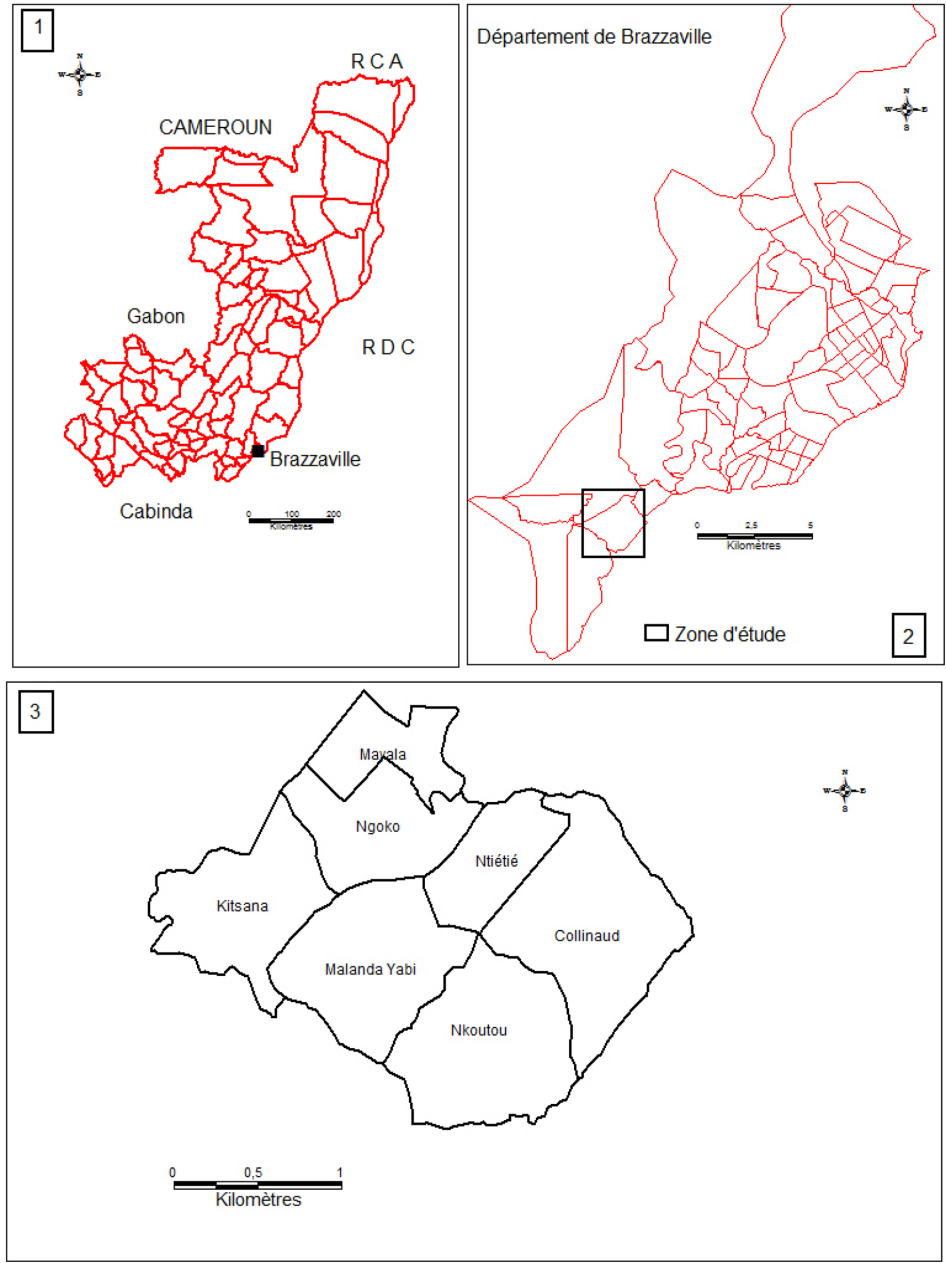
Maps of the study zone. *1* Republic of Congo; *2* map of Brazzaville and the location of study zone; *3* details of study zone (local subdivisions)

The study was performed from April 2010 to March 2011 in Madibou and Mbouono, two suburban areas located in Makélékélé, one of the seven districts of Brazzaville. According to the data obtained from the General Census of Population in 2007, there were 1,373,382 inhabitants in Brazzaville, and Makélékélé, one of its seven districts, had 298,292 inhabitants. Makélékélé is largely covered by vegetation and crossed by several streams that provide sources of water for agricultural activities (Fig. 1). In this study area, Ngoko, Kinsana, and Mayala are part of Madibou; Ntietie, Malanda Yabi, Collinaud, and Nkoutou are part of Mbouono. A public health facility is located in Madibou which refers some patients to the district hospital in Makélékélé, located in the centre of the district. In the 1980s, malaria transmission had been described as intense and perennial, with an entomological inoculation rate of 200–1000 infective bites/person/year [24]. Since then, there has been no update with more recent entomological studies.

The study population was a cohort of 330 children under 10 years, established after a population census. Of 17,636 inhabitants in the study area, 3058 (17.3 %) were children under 10 years of age.

### Patients

Children were examined and passively followed for febrile illnesses at the parents’ request by a medical doctor in the medical unit established at the Makélékélé district hospital, located about 6 km from the study area. Fingerprick capillary blood was obtained to prepare Giemsa-stained smears for malaria parasite examination by microscopy. After obtaining informed consent from one of the parents, venous blood sample was collected from children with positive smears for *P. falciparum* malaria parasites in ethylene diaminetetraacetic acid (EDTA)-coated tubes to determine haematological parameters using Beckman Coulter ACT Diff (Beckman Coulter; Brea, CA, USA).

Febrile children with positive smears were randomized to receive ASAQ or AL if the following conditions were met: monoinfection with *P. falciparum*, parasitaemia ≥1000 asexual parasites per microlitre of blood, absence of severe malnutrition, danger signs (i.e., inability to stand, breastfeed, or drink, recent convulsions, lethargy, or persistent vomiting), signs of severe and complicated malaria, and any febrile conditions due to diseases other than malaria [25].

### Treatment

In the present randomized, open-label study, patients were assigned to one of the treatment groups using a computer-generated table of random numbers prepared in advance by a statistician. Drugs were administered for three consecutive days (day 0, 1, and 2). ASAQ (Coarsucam^®^, Sanofi-Aventis, Antony, France) treatment group received standard once-daily doses based on body weight. ASAQ was administered with water. AL (Coartem^®^, Novartis Pharma, Basel, Switzerland) was administered with milk, two doses/day, for 3 days. All ASAQ doses were administered under the supervision of a nurse. Only the first, third, and fifth doses of AL were supervised during daily consultation. The second, fourth, and sixth doses of AL were given to parents for administration at home at a specified time. A supply of powdered milk was provided to the parent or guardian for drug administration. As the medical unit conducting the present study was far from the residential area, the phone number of parents was recorded to remind them to administer the second dose eight hours after the first dose, as well as before the fourth and sixth doses.

After drug administration, the patient was observed for 30 min to record any vomiting which required re-treatment. A second vomiting resulted in the exclusion of children from the drug evaluation protocol and administration of artesunate by intramuscular route. Three doses of paracetamol were provided to the parents for fever management at home.

### Follow-up

Patients were followed on an outpatient basis on days 1, 2, 3, 7, 14, 21 and 28 according to the 2009 WHO protocol [25]. If the patients were absent on the appointment day, the study team visited their homes. From day 2 to day 28, two thick films were made for the systematic control of parasitaemia. However, in case of high fever or danger signs on day 1, thick smears were prepared and examined immediately. From day 14, fingerprick capillary blood was collected on Whatman 3MM filter papers, dried for 24 h at 37 °C, sealed in plastic bags, and stored in a dry place.

Fever with negative smears was routinely treated with paracetamol and amoxycilline when an antibiotic was needed. If an antibiotic with anti-malarial activity was prescribed by the medical staff, the child was excluded from the assessment of anti-malarial drug efficacy, but was still followed, as part of the cohort.

If recrudescent malaria parasites were diagnosed, children initially treated with ASAQ at inclusion received AL as the second-line treatment, while those who were initially treated with AL at inclusion received ASAQ. These children were followed for additional 28 days.

### Drug safety and tolerability

From day 1 to day 28, adverse events were assessed indirectly by questioning the parents or actively by clinical exploration to detect any symptoms or signs that were absent on day 0. An adverse event was defined as a sign, symptom, or illness, which was absent on day 0 but occurred during follow-up. All adverse events were recorded in individual case record file.

### Malaria diagnosis and haematological and biochemical analyses

Giemsa-stained thick films were examined under the microscope. Asexual parasites were counted against 200 white blood cells (WBCs) and expressed as the number of asexual parasites/μL of blood, assuming a WBC count of 8000/μL of blood. In case of hyperparasitaemia, the parasite count was determined when 500 asexual parasites were counted even if 200 WBCs were not reached. Parasite density was determined by two independent technicians. Additional thick films were prepared from venous blood and stained 24 h later. The results of both blood examinations were compared and expressed as the mean of two results.

### Malaria parasite genotyping

DNA was extracted from Whatman 3MM filter papers by the Chelex method [26]. As recommended by the WHO [27], pre-treatment and post-treatment isolates were compared sequentially by nested PCR using merozoite surface protein-2 (*msp2*), merozoite surface protein-1 (*msp1*), and glutamine-rich protein (*glurp*) polymorphic genetic markers. Primary PCR was performed to amplify fragments of the markers with primers hybridizing within conserved regions. Secondary nested PCR was performed with internal primers specific to allelic families: 3D7 and FC27 for *msp2* and K1, MAD20, and RO33 for *msp1*. PCR products were visualized by fluorescent end-labelling of internal primers. After capillary electrophoretic separation with automated DNA sequencer ABI310 (Applied Biosystems), allele size and peak heights for individual alleles were measured using GeneScan software (Applied Biosystems). Minor peaks <25 % of the amplitude of the predominant allele and poorly amplified loci with <200 fluorescence units were discarded. Recrudescence was defined as the presence of at least one identical size allele in all three markers (or the presence of some, or all, identical size alleles in case of multiple alleles). Reinfection was defined as the presence of different size allele (or the presence of all alleles of different sizes, in case of multiple alleles) in one of the three markers.

### Haemoglobin genotyping

Genomic human DNA was extracted from 200 μL of whole blood sample using Qiaamp DNA blood mini kit (Qiagen, Hilden, Germany) according to the manufacturer’s instruction. DNA was recovered in 200 μL of elution buffer provided in the kit and stored at −20 °C until use.

The polymorphism in the β-chain of the globin gene at codon six was determined by using an allele-specific PCR method [28, 29]. The PCR conditions were as follows: one cycle of 5 min at 94 °C, 25 cycles of 94 °C for 1 min, 55 °C for 2 min, and 72 °C for 3 min. As quality control, two PCR amplifications were performed for each sample for A and S gene detection. The expected fragment length was 203 bp. DNA samples extracted from individuals with known AA, AS and SS haemoglobin were used as controls. PCR products were analysed by electrophoresis in a 1.5 % agarose gel.

### Treatment outcomes

The primary end-points were PCR-unadjusted and PCR-adjusted cure rates on day 28. Clinical outcome was classified as early treatment failure (ETF), late clinical failure (LCF), late parasitological failure (LPF), adequate clinical and parasitological response (ACPR), and proportion of new infections after PCR analysis of blood samples from patients with recurrent parasitaemia. The second end-points were proportions of treated patients with fever and parasitaemia on day 2 and day 3, and adverse events from day 1 to day 7.

### Statistical analyses

The number of recruited patients with uncomplicated malaria from the cohort was based on the assumption of 0.8–0.9 uncomplicated malaria episode/child/year, which would allow recruitment of 260–300 malaria-infected children randomly assigned to one of the two treatment arms, ASAQ and AL, in a year. Per-protocol (PP) data were analysed using a pre-programmed Excel 7 spreadsheet provided by the Department of Global Malaria Programme, WHO (Geneva, Switzerland). This analysis method allows a direct comparison of data of the present study with those of previous studies [17, 18].

The Chi square test was used to compare proportions and analyse the degree of association between drug efficacy and haemoglobin genotype (AA or AS). Quantitative variables were compared by the analysis of variance. The 95 % confidence interval (95 % CI) of percentages was calculated using the exact binomial test. The level of significance (*P*) was fixed at 0.05 for all statistical tests.

### Ethical approval

The present study was reviewed and approved by the institutional ethics committee of research in health science (CERSSA) of the Congolese Ministry of Research and the Ministry of Health and Population of RoC. The study was conducted in accordance with the ethical principles of the Declaration of Helsinki. The community was informed through meetings with community leaders, posters, and banners. Before children were recruited in the cohort, parents were informed about the objectives of the project, benefits, and constraints associated with participation in the study. Parents or guardians who agreed to participate in the study signed the informed consent form in French or in one of the two national languages.

## Results

### Patient characteristics

**Fig. 2:**
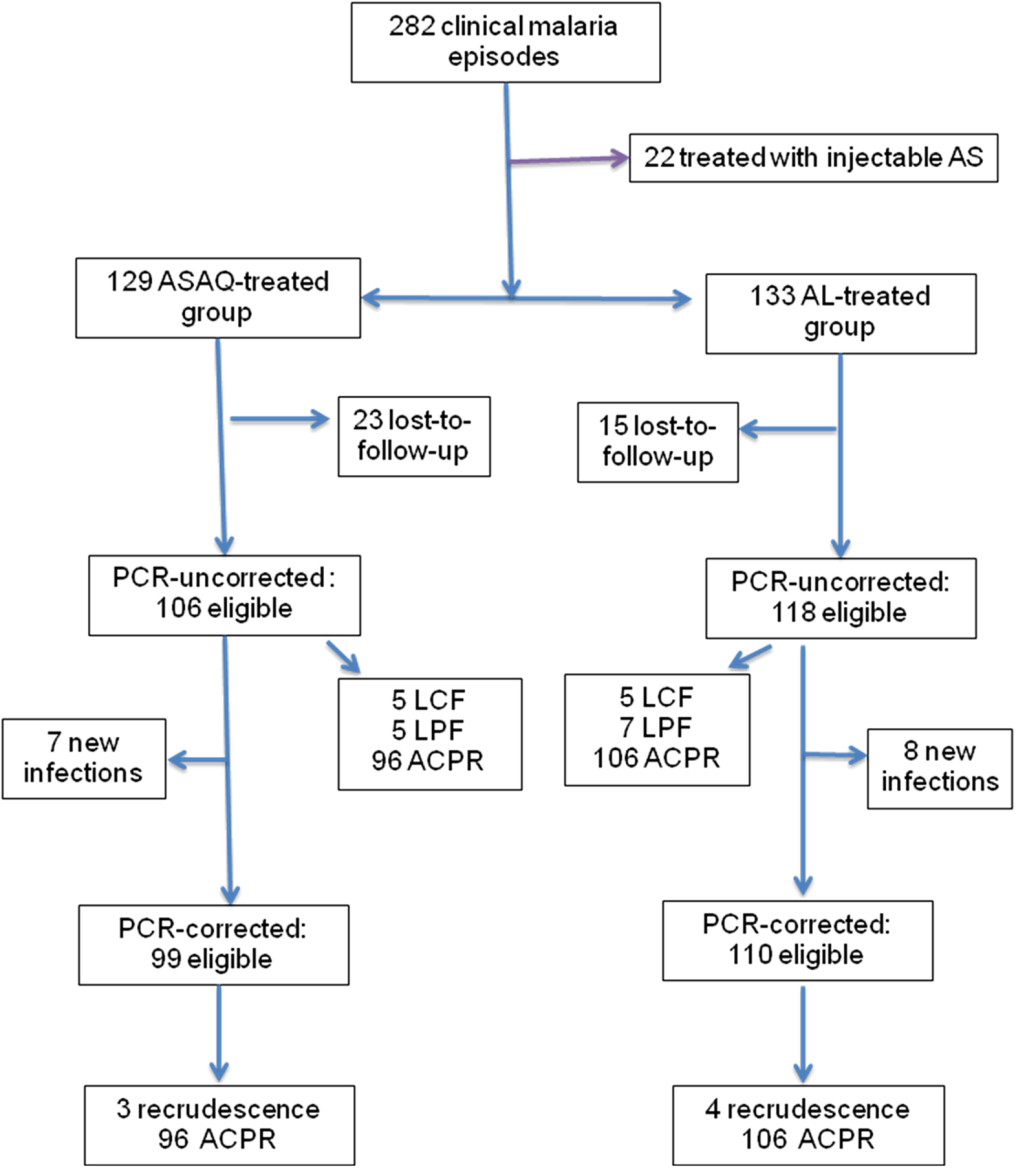
Enrolment and follow-up profile

From April 2010 to March 2011, 282 malaria episodes were registered during passive follow-up. Of these episodes, 262 were included in drug efficacy study: 129 were randomized to ASAQ group, while 133 were assigned to AL group (Fig. 2). Twenty children with malaria episode had extreme fatigue and/or repeated vomiting and were not included in the treatment protocol. They were referred to physicians in the paediatric ward of the district hospital of Makélékélé for treatment with parenteral artesunate.

In the ASAQ treatment group, 52 (40.3 %) children were under 5 years of age, while in the AL group 54 (40.6 %) were under 5 years of age. Thirty-three patients with parasite density >200,000 asexual parasites/µL, but without danger signs of severe and complicated malaria, were included [15 (8 under 5 years of age) in the ASAQ group and 18 (8 under 5 years of age) in the AL group].

There were 112 (86.8 %) and 109 (82.0 %) AA haemoglobin genotype in ASAQ and AL treatment groups, respectively (*P* = 0.6). There were 17 of 129 (13.2 %) children and 24 of 133 (18.0 %) children with AS in the ASAQ and AL groups, respectively. The baseline characteristics of enrolled patients in each group were similar, with the exception of the mean geometric parasite density (*P* < 0.001) (Table 1). Among ASAQ and AL groups, 23 (17.8 %) and 15 (12.7 %) patients were lost to follow-up on or before day 28, despite home visits.

**Table 1:** Basic clinical and parasitological characteristics of patients in the cohort with malarial episodes

	ASAQ	AL	P
Number of patients	129	133	
Age (months), mean (±SD)	64.9 ± 33.0	65.0 ± 30.6	1
Sex: female/male	42/70	57/76	0.09
Weight (kg), mean (±SD)	19.8 ± 6.3	19.6 ± 5.6	0.8
Axillary temperature (°C), mean (±SD)	38.0 ± 1.0	37.6 ± 1.3	0.5
GMPD, asexual parasites/µL	29,100	30,700	0.5
Range	950–388,000	800–725,000	
PD <1000, N (%)	4 (3.1)	3 (2.3)	1
PD 1000–200,000, N (%)	110 (85.3)	112 (84.2)	0.7
PD ≥200,000, N (%)	15 (11.6)	18 (13.5)	0.5
Mean haemoglobin, g/dL (±SD)	10.6 (1.1)	10.5 (1.3)	0.5
Anaemia (Hb < 11 g/dL), N (%)	75 (58.1 %)	78 (58.6)	0.9
AA genotype, N (%)	112 (86.8)	109 (82.0)	0.4
GMPD, asexual parasites/µL	29,500	30,300	0.8
Range	950–376,000	800–725,000	
AS genotype, N (%)	17 (13.2)	24 (18.0)	0.3
GMPD, asexual parasites/µL	19,000	39,300	0.0001
Range	950–388,000	3360–487,000	

Anaemia was defined as haemoglobin (Hb) <11 g/dL

*GMPD* geometric mean parasite density, *PD* parasite density

### Treatment outcomes

#### Cure rates on day 28

The PCR-uncorrected cure rates were 90.6 and 89.8 % in ASAQ and AL groups. Taking into account haemoglobin genotype, the cure rates in AA carriers treated with ASAQ or AL were 90.0 and 88.6 %, respectively, while in AS patients, the cure rates for ASAQ and AL were 93.8 and 90.9 %, respectively (Table 2). After PCR adjustment, the overall cure rates in ASAQ- and AL-treated children were 97.0 and 96.4 %, respectively. In AA children, the cure rates for ASAQ and AL were 96.4 and 96.7 %, respectively, while in AS carriers treated with ASAQ and AL, the cure rates were 100 and 95.2 %, respectively.

**Table 2:** Treatment outcomes of ASAQ and AL treatment, per-protocol analysis

	ASAQ			AL		
	Overall	AA	AS	Overall	AA	AS
Number of patients, N	129	112	17	133	109	24
PCR uncorrected responses on day 28						
Lost to follow-up, N (%)	22 (17.1)	22 (19.6)	1 (5.9)	15 (11.3)	13 (11.9)	2 (8.3)
Eligible, N (%)	107 (82.9)	90 (80.4)	16 (94.1)	118 (88.7)	96 (88.1)	22 (91.7)
Failure, N (%)	10 (9.4)	9 (10.0)	1 (6.2)	12 (10.2)	10 (10.4)	2 (9.1)
ETF, N (%)	0	0	0	0	0	0
LCF, N (%)	5 (4.7)	4 (4.4)	1 (6.2)	5 (4.2)	5 (5.2)	0
LPF, N (%)	5 (4.7)	5 (5.6)	0	7 (5.9)	5 (5.2)	2 (9.1)
ACPR, N (%)	97 (90.6)	81 (90.0)	15 (93.8)	106 (89.8)	86 (89.6)	20 (90.9)
PCR corrected responses on day 28						
Loss of follow up + new infection, N (%)	30 (23.3)	28 (25.0)	2 (11.8)	23 (17.3)	20 (18.3)	3 (12.5)
Eligible, N (%)	99 (76.7)	84 (75.0)	15 (88.2)	110 (82.7)	89 (81.7)	21 (87.5)
Failure, N (%)	3 (3.0)	3 (3.6)	0	4 (3.6)	3 (3.3)	1 (4.8)
ETF, N (%)	0	0	0	0	0	0
ACPR, N (%)	96 (97.0)	81 (96.4)	15 (100)	106 (96.4)	86 (96.6)	20 (95.2)
Recrudescence, N	3	3	0	4	3	1
New infection, N	7	6	1	8	7	1

*ASAQ* artesunate-amodiaquine, *AL* artemether-lumefantrine, *ETF* early treatment failure, *LCF* late clinical failure, *LPF* late parasitological failure, *ACPR* adequate clinical and parasitological response

Among children under 5 years of age, the PCR-corrected cure rate was 97.3 % in the ASAQ group and 95.2 % in the AL group. High parasitaemia did not influence the efficacy outcomes. Among 15 ASAQ-treated and 18 AL-treated children with day 0 parasite density more than 200,000 asexual parasites/µL, one recrudescence was recorded in each group treatment, while in patients with 1000–200,000 asexual parasites/µL, two and three recrudescent infections were observed in ASAQ and AL groups, respectively.

In the ASAQ treatment group, 78/129 (60.5 %) children received this drug during their first malaria episode, 51/129 (39.5 %) during their second, third or fourth malaria episodes (Table 3). Considering the first episode treated with ASAQ, 59/60 (98.3 %) had therapeutic success and 1/60 (1.7 %) had treatment failure. There were three new infections and 15 patients lost to follow-up. Of 51 patients previously treated with AL during the first episode, 37/39 (94.9 %) and 2/39 (5.1 %) responded with treatment success and treatment failure, respectively. In this group, five new infections and seven lost to follow-up were registered.

**Table 3:** Efficacy of ASAQ and AL as first-line and second-line treatments

	First treated malaria episode	≥Second treated malaria episode	
ASAQ			
ACPR, N (%)	59 (98.3)	37 (94.9)	RR = 1.04 (0.96–1.12)
Recrudescence, N (%)	1 (1.7)	2 (5.1)	P = 0.3
New infection	3	5	
Lost-to-follow-up	15	7	
AL			
ACPR, N (%)	69 (94.5)	37 (100.0)	RR = 0.95 (0.89–1.00)
Recrudescence, N (%)	4 (5.5)	0	P = 0.19
New infection	4	4	
Lost-to-follow-up	10	5	
	RR = 1.04 (0.98–1.11), P = 0.2	RR = 0.95 (0.88–1.02), P = 0.26	

*ASAQ* artesunate-amodiaquine, *AL* artemether-lumefantrine, *ACPR* adequate clinical and parasitological response

In the AL treatment group, 87/133 (65.4 %) received this drug during the first malaria episode, of which 69 (94.5 %) were successfully treated (ACPR), four (5.5 %) failures, four new infections, and 10 lost-to-follow-up (Table 3). Of 46 patients (34.6 %) who had been treated previously with ASAQ during their first episode, 37/37 (100 %) were therapeutic success, four were new infections, and five were lost to follow-up.

#### Fever and parasites clearance from day 0 to day 3

Among patients assigned to ASAQ treatment group, 94, 98 and 97 % became afebrile on day 1, 2, and 3, respectively. Among patients in AL group, 91, 96, and 97 % were afebrile on day 1, 2, and 3, respectively. On day 2 and day 3, the number of patients with positive smears and parasitaemia decreased considerably. In the ASAQ treatment group, 5 of 130 patients (3.8 %) still had positive smears on day 2, with a geometric mean of 87 asexual parasites/µL. On day 3, none of the patients followed (n = 121) had a positive smear.

#### Treatment safety

Between day 0 and day 7, a total of 166 and 111 adverse events were reported by patients treated with ASAQ and AL, respectively (Table 4). Asthenia was the most commonly reported adverse event and was more frequent in the ASAQ group (*P* = 0.02). This side effect was followed by vomiting, abdominal pain, diarrhoea, headache, jaundice, and dizziness, with similar frequencies. Pruritus occurred in four ASAQ treated children, while only one AL treated child reported pruritus. Rashes appeared in one ASAQ treated child.

**Table 4:** Clinical adverse events in the per protocol population

	ASAQ (number of patients)							AL (number of patients)							P
	D0	D1	D2	D3	D7	Total	%	D0	D1	D2	D3	D7	Total	%	
*Adverse effects*	129	125	123	119	114	–		133	132	130	125	126	–		
Fatigue	2	18	19	25	1	65	10.7	3	14	12	5	4	38	5.9	0.02
Anorexia	3	1	1	2	2	9	1.5	0	1	1	2	1	5	0.8	0.2
Vomiting	8	10	4	4	2	28	4.6	11	9	5	0	1	26	4.0	0.6
Abdominal pain	3	6	4	4	2	19	3.1	2	2	2	3	5	14	2.2	0.3
Diarrhoea	2	3	4	0	2	11	1.8	9	2	1	0	0	12	1.9	0.9
Nausea	0	3	1	2	0	6	1	0	0	1	0	1	2	0.3	0.2
Headache	3	3	1	0	2	9	1.5	0	2	2	2	2	8	1.2	0.7
Jaundice	0	1	0	2	4	7	1.1	1	0	0	1	1	3	0.5	0.3
Dizziness	0	1	3	3	0	7	1.1	0	0	1	1	0	2	0.3	0.1
Pruritus	0	0	2	1	1	4	0.7	0	0	1	0	0	1	0.2	0.3
Rashes	0	0	0	0	1	1	0.2	0	0	0	0	0	0	0	–

*ASAQ* artesunate-amodiaquine, *AL* artemether-lumefantrine

Dizziness, nausea, headache and jaundice were reported more frequently (P < 0.05) in children aged 5–10 years in both ASAQ and AL treatment groups. Skin manifestations (rash, pruritus) were mostly observed in under-five children in both treatment groups. The difference in the frequency of fatigue, vomiting, and abdominal pain was not significantly different (P > 0.05) between the age groups <5 years and 5–10 years old.

## Discussion

This is the second study on ASAQ and AL efficacy and safety in Brazzaville, the capital city of RoC. Under 10 years children of the cohort with uncomplicated falciparum malaria were randomly treated with ASAQ or AL. The presence of sickle cells was determined to assess if they influence ACT efficacy in children.

All children recruited in the cohort reside in the suburban area in the south of Brazzaville. Despite the impact of urbanization, malaria transmission is still intense in this area where children who are frequently infected with high parasite loads without signs of severe malaria are usually treated in health facilities for uncomplicated malaria. In the present cohort, high parasitaemias (>200,000 asexual parasites/µL) were similarly distributed in under-five children and those between 5 and 10 years old.

Data of this randomized study confirm the high and comparable efficacy of these two forms of ACT in Brazzaville, confirming previous non-randomized trials conducted from 2005 to 2006 [17, 18]. Data were compared based on per protocol analysis. As in the previous studies, ASAQ treatment was fully supervised, but AL administration was partially supervised, as in many other studies conducted in Africa. While ASAQ was non-co-formulated combination in the study conducted in 2005–2006, patients received the standard dose (quarter, half, or three-quarters of amodiaquine and artesunate tablets) according to their body weight [18]. In the present study, co-formulated ASAQ was administered according to manufacturer’s instructions. The PCR-corrected ASAQ efficacy in the previous study was 94.4 % among 151 patients while in the present study the efficacy was 97.0 % (n = 97 patients, p = 0.6). The PCR-corrected AL efficacy was 97.1 % in 67 patients in the previous study [17]. By comparison, AL efficacy was 96.4 % in 106 patients included in the present study (p = 0.9).

Treatment was systematically switched after a previous uncomplicated malaria episode: the patient was treated with AL if he or she had been previously treated with ASAQ, and the patient was treated with ASAQ, if the previous treatment had been AL. Both combinations for the first- and second-line malaria management had a comparable efficacy. In accordance with the Congolese national policy, ASAQ treatment failures were treated with AL. These results suggest that in public health centres either ASAQ or AL can be used as the first-line drug and offer the possibility to use AL to treat patients who are reluctant to take ASAQ due to side effects or past history of allergic reactions. In the early 2000s, sub-Saharan African countries started to implement the new anti-malarial drug policy based on ACT [11]. Among 43 sub-Saharan countries, 15 adopted ASAQ, 22 AL, and 2 both ASAQ and AL [30]. At that time, the availability of ACT was the main criterion of choice. Drug efficacy studies had not been carried out prior to the adoption of new policies in most countries. In 2014, RoC modified the national anti-malarial drug policy: AL for the first-line treatment and ASAQ as the second-line drug. The main reason advanced by the National Malaria Control Programme was that ASAQ is associated with more side effects than AL [31]. However, there has been no pharmacovigilance study or studies on the acceptability of ASAQ and AL.

In this study the percentage of patients with at least one side effect associated with ASAQ intake was higher than that of patients treated with AL. In particular, asthenia was reported more frequently after ASAQ treatment. Previous studies have reported that patients receiving AQ monotherapy or AQ in combination with another anti-malarial drug complained of fatigue, abdominal pain, and vomiting [32, 33]. In Democratic Republic of Congo (DR Congo), anorexia and physical weakness were more commonly observed after ASAQ treatment, as compared with AL [34]. In recent years, pharmacies in Congo offer a wide range of AL specialties, including specialties for adults and dispersible formulations for young children. Pharmaceutical firms are also promoting AL specialties. These factors are expected to favour a pronounced increase in the use of and reliance on AL to treat uncomplicated malaria in the country.

Sickle cell disease is the most common genetic disease in sub-Saharan African countries. It was estimated that in 2010 the world recorded 305,800 newborns with sickle cell trait, with DR Congo (140,800) and Nigeria (91,000) being the most affected [35]. In Gabonese hospitals, 16.3 and 1.4 % of newborns were carriers of AS and SS genotypes, respectively; in the DR Congo, there were 17.6 and 1.2 % of newborns with AS and SS genotypes, respectively [36]. Sickle cell trait is, therefore, widely present in African countries which carry much of malaria burden in the world [37]. The heterozygous genotype AS is also known to provide protection against *P. falciparum* malaria [38–41]. It is, therefore, of interest to investigate the effect of sickle cell trait on ACT efficacy. For intermittent preventive treatment of malaria in children, studies have shown that sulfadoxine-pyrimethamine (SP) is more effective than CQ in paediatric carriers of HbAS [42]. Moreover, when treated with SP, twice as many HbAA children had failure than those with HbAS [20]. The present study has shown that ASAQ and AL have a comparable efficacy in malaria-infected children with HbAA and HbAS.

## Conclusion

This first randomized study in Brazzaville, conducted 6 years after the change of policy against malaria and 6 years after the first ASAQ and AL non-randomized studies, confirms the high efficacy of these combinations, both of which are currently used in co-formulation whereas in 2006, only AL was co-formulated. ACT efficacy in patients carrying AA and AS haemoglobin allows the use of these drugs regardless of sickle cell trait. The recurring problem of adverse events associated with ASAQ warrants further studies on the acceptability of both ACT.

## References

[1] Mabiala-Babela JR, Makoumbou PB, Mbika Cardorelle A, Tsiba JB, Senga P (2009). Evolution de la mortalité hospitalière chez l’enfant à Brazzaville. Med Afr Noire.

[2] Moyen G, Nzingoula S, Mowandza-Ndinga JC, Nkoua JL, Mpemba AB, Fourcarde V (1073). Le paludisme de l’enfant dans un service de pédiatrie à Brazzaville. A propos de 1073 observations. Méd Afr Noire.

[3] Ndounga M, Casimiro PN, Miakassissa-Mpassi V, Loumouamou D, Ntoumi F, Basco LK (2008). Malaria in health centres in the southern districts of Brazzaville, Congo. Bull Soc Pathol Exot..

[4] Mayengue PI, Ndounga M, Matondo Maya D, Ntandou N, Ntoumi F (2005). In vivo chloroquine resistance of the pfcrt codon 76 mutation in *Plasmodium falciparum* isolates from the Republic of Congo. Acta Trop..

[5] Ndounga M, Mayengue PI, Tahar R, Casimiro PN, Matondo Maya DW, Miakassissa-Mpassi V (2007). Efficacy of sulfadoxine-pyrimethamine, amodiaquine, and sulfadoxine-pyrimethamine-amodiaquine combination for the treatment of uncomplicated falciparum malaria in the urban and suburban areas of Brazzaville (Congo). Acta Trop.

[6] Nsimba B, Jafari-Guemouri S, Malonga DA, Mouata AM, Kiori J, Louya F (2005). Epidemiology of drug-resistant malaria in Republic of Congo: using molecular evidence for monitoring antimalarial drug resistance combined with assessment of antimalarial drug use. Trop Med Int Health..

[7] Ndounga M, Tahar R, Basco LK, Casimiro PN, Malonga DA, Ntoumi F (2007). Therapeutic efficacy of sulfadoxine-pyrimethamine and the prevalence of molecular markers of resistance in under 5-year olds in Brazzaville, Congo. Trop Med Int Health..

[8] Ndounga M, Ebata-Mongo S, Mallandah G, Bassouamina L, Issoibeka R, Basco L (2002). Fièvres et prise en charge du paludisme dans les centres de santé intégré de Brazzaville (Congo). Bulletin de Liaison et de Documentation de l’OCEAC.

[9] WHO (2001). Antimalarial drug combination therapy. Report of a WHO technical consultation.

[10] WHO (2006). Guidelines for the treatment of malaria.

[11] WHO (2006). The African malaria report.

[12] WHO (2012). World Health Report 2012.

[13] D’Acremont V, Lengeler C, Genton B (2010). Reduction in the proportion of fevers associated with *Plasmodium falciparum* parasitaemia in Africa: a systematic review. Malar J..

[14] O’Meara WP, Mangeni JN, Steketee R, Greenwood B (2010). Changes in the burden of malaria in sub-Saharan Africa. Lancet Infect Dis..

[15] Ministère de la Santé et de la Population (2006). République du Congo: *Politique nationale de lutte contre le paludisme*.

[16] van den Broek I, Kitz C, Al Attas S, Libama F, Balasegaram M, Guthmann JP (2006). Efficacy of three artemisinin combination therapies for the treatment of uncomplicated *Plasmodium falciparum* malaria in the Republic of Congo. Malar J.

[17] Ndounga M, Tahar R, Casimiro PN, Loumouamou D, Basco LK (2012). Clinical efficacy of artemether-lumefantrine in Congolese children with acute uncomplicated *falciparum* malaria in Brazzaville. Malar Res Treat..

[18] Ndounga M, Mayengue PI, Casimiro PN, Loumouamou D, Basco LK, Ntoumi F (2013). Artesunate-amodiaquine efficacy in Congolese children with acute uncomplicated *falciparum* malaria in Brazzaville. Malar J.

[19] White NJ (1998). Why is it that antimalarial drug treatments do not always work. Ann Trop Med Parasitol.

[20] Terlouw DJ, Aidoo MA, Udhayakumar V, Kolczak MS, Oloo AJ, Kager PA (2002). Increased efficacy of sulfadoxine-pyrimethamine in the treatment of uncomplicated falciparum malaria among children with sickle cell trait in Western Kenya. J Infect Dis.

[21] Yuthavong Y, Butthep P, Bunyaratvej A, Fucharoen S (1989). Decreased sensitivity of artesunate and chloroquine of *Plasmodium falciparum* infecting hemoglobin H and/or hemoglobin constant spring erythrocytes. J Clin Invest..

[22] Senok AC, Nelson EAS, Li K, Oppenheimer SJ (1997). Thalassaemia trait, red blood cell age and oxidant stress: effects on growth and sensitivity to artemisinin. Trans R Soc Trop Med Hyg.

[23] Mockenhaupt FP, May J, Bergqvist Y, Meyer CG, Falusi AG, Bienzle U (2001). Evidence for a reduced effect of chloroquine against *Plasmodium falciparum* in alpha-thalassaemic children. Trop Med Int Health.

[24] Trape JF, Zoulani A (1987). Malaria and urbanization in central Africa: the example of Brazzaville. Part II: Results of entomological surveys and epidemiological analysis. Trans R Soc Trop Med Hyg.

[25] WHO (2009). Methods for surveillance of antimalarial drug efficacy.

[26] Kain KC, Lanar DE (1991). Determination of genetic variation within *Plasmodium falciparum* by using enzymatically amplified DNA from filter paper disks impregnated with whole blood. J Clin Microbiol.

[27] WHO (2008). Methods and techniques for clinical trials on antimalarial drug efficacy: genotyping to identify parasite populations.

[28] Wu DY, Ugozzoli L, Pal BK, Wallace RB (1989). Allele-specific enzymatic amplification of beta-globin genomic DNA for diagnosis of sickle cell anemia. Proc Natl Acad Sci USA.

[29] Matondo Maya DW, Mavoungou E, Deloron P, Theisen M, Ntoumi F (2006). Distribution of IgG subclass antibodies specific for *Plasmodium falciparum* glutamate-rich-protein molecule in sickle cell trait children with asymptomatic infections. Exp Parasitol..

[30] WHO (2008). World malaria report 2008.

[31] Ministère de la santé et de la Population. Politique nationale de lutte contre le paludisme. République du Congo; 2014.

[32] Djallé D, Njuimo SP, Manirakiza A, Laganier R, Le Faou A, Rogier C (2014). Efficacy and safety of artemether + lumefantrine, artesunate + sulphamethoxypyrazine-pyrimethamine and artesunate + amodiaquine and sulphadoxine-pyrimethamine + amodiaquine in the treatment of uncomplicated falciparum malaria in Bangui, Central African Republic: a randomized trial. Malar J..

[33] Ndayiragije A, Niyungeko D, Karenzo J, Niyungeko E, Barutwanayo M, Ciza A (2004). Efficacy of therapeutic combinations with artemisinin derivatives in the treatment of non complicated malaria in Burundi. Trop Med Int Health.

[34] Onyamboko MA, Fanello CI, Wongsaen K, Tarning J, Cheah PY, Tshefu KA (2014). Randomized comparison of the efficacies and tolerabilities of three artemisinin-based combination treatments for children with acute *Plasmodium falciparum* malaria in the Democratic Republic of the Congo. Antimicrob Agents Chemother.

[35] Piel FB, Hay SI, Gupta S, Weatherall DJ, Williams TN (2013). Global burden of sickle cell anaemia in children under five, 2010-2050: modelling based on demographics, excess mortality, and interventions. PLoS Med..

[36] Ngasia B (2010). Proceedings of the first symposium on sickle cell disease in Central Africa. Med Trop (Mars)..

[37] WHO (2014). World Malaria report 2014.

[38] Aidoo M, Terlouw DJ, Kolczak MS, McElroy PD, ter Kuile FO, Kariuki S (2002). Protective effects of the sickle cell gene against malaria morbidity and mortality. Lancet.

[39] Williams TN (2006). Human red blood cell polymorphisms and malaria. Curr Opin Microbiol.

[40] Eridani S (2011). Sickle cell protection from malaria. Hematol Rep..

[41] Ferreira A, Marguti I, Bechmann I, Jeney V, Chora A, Palha NR, Rebelo S, Henri A, Beuzard Y, Soares MP (2011). Sickle hemoglobin confers tolerance to *Plasmodium* infection. Cell.

[42] Nakibuuka V, Ndeezi G, Nakiboneka D, Ndugwa CM, Tumwine JK (2009). Presumptive treatment with sulphadoxine-pyrimethamine versus weekly chloroquine for malaria prophylaxis in children with sickle cell anaemia in Uganda: a randomized controlled trial. Malar J..

